# Screening of cancer tissue arrays identifies CXCR4 on adrenocortical carcinoma: correlates with expression and quantification on metastases using ^64^Cu-plerixafor PET

**DOI:** 10.18632/oncotarget.19945

**Published:** 2017-08-04

**Authors:** Ido D. Weiss, Lyn M. Huff, Moses O. Evbuomwan, Xin Xu, Hong Duc Dang, Daniel S. Velez, Satya P. Singh, Hongwei H. Zhang, Paul J. Gardina, Jae-Ho Lee, Liza Lindenberg, Timothy G. Myers, Chang H. Paik, David S. Schrump, Stefania Pittaluga, Peter L. Choyke, Tito Fojo, Joshua M. Farber

**Affiliations:** ^1^ Laboratory of Molecular Immunology, National Institute of Allergy and Infectious Diseases, National Institutes of Health, Bethesda, MD, USA; ^2^ Medical Oncology Branch, Center for Cancer Research, National Cancer Institute, National Institutes of Health, Bethesda, MD, USA; ^3^ Laboratory of Pathology, Center for Cancer Research, National Cancer Institute, National Institutes of Health, Bethesda, MD, USA; ^4^ Genomic Technologies Section, Research Technologies Branch, National Institute of Allergy and Infectious Diseases, National Institutes of Health, Bethesda, MD, USA; ^5^ Radiopharmaceutical Laboratory, Nuclear Medicine Division, Radiology and Imaging Sciences, Clinical Center, National Institutes of Health, Bethesda, MD, USA; ^6^ Molecular Imaging Program, Center for Cancer Research, National Cancer Institute, National Institutes of Health, Bethesda, MD, USA; ^7^ Thoracic Epigenetics Section, Thoracic and GI Oncology Branch, Center for Cancer Research, National Cancer Institute, National Institutes of Health, Bethesda, MD, USA

**Keywords:** CXCR4, cancer, PET, plerixafor, adrenal

## Abstract

Expression of the chemokine receptor CXCR4 by many cancers correlates with aggressive clinical behavior. As part of the initial studies in a project whose goal was to quantify CXCR4 expression on cancers non-invasively, we examined CXCR4 expression in cancer samples by immunohistochemistry using a validated anti-CXCR4 antibody. Among solid tumors, we found expression of CXCR4 on significant percentages of major types of kidney, lung, and pancreatic adenocarcinomas, and, notably, on metastases of clear cell renal cell carcinoma and squamous cell carcinoma of the lung. We found particularly high expression of CXCR4 on adrenocortical cancer (ACC) metastases. Microarrays of ACC metastases revealed correlations between expression of *CXCR4* and other chemokine system genes, particularly *CXCR7/ACKR3*, which encodes an atypical chemokine receptor that shares a ligand, CXCL12, with CXCR4. A first-in-human study using ^64^Cu-plerixafor for PET in an ACC patient prior to resection of metastases showed heterogeneity among metastatic nodules and good correlations among PET SUVs, CXCR4 staining, and *CXCR4* mRNA. Additionally, we were able to show that CXCR4 expression correlated with the rates of growth of the pulmonary lesions in this patient. Further studies are needed to understand better the role of CXCR4 in ACC and whether targeting it may be beneficial. In this regard, non-invasive methods for assessing CXCR4 expression, such as PET using ^64^Cu-plerixafor, should be important investigative tools.

## INTRODUCTION

CXCR4 is a chemokine receptor, a member of a subfamily of twenty G-protein coupled chemoattractant receptors that mediate leukocyte trafficking. CXCR4’s sole known chemokine agonist is CXCL12. CXCR4 is unusual among chemokine receptors in that it plays fundamental roles in the hematopoietic, cardiovascular, reproductive, and nervous systems during embryonic development [1]. Its role in cancer has been investigated for fifteen years with expression reported in hematologic malignancies, breast, ovarian, cervical, gastric, colorectal, pancreatic, prostate, lung, and renal (clear cell) carcinomas, as well as sarcomas [2-7]. Depending on the tumor type, expression of CXCR4 has been reported in 20%-80% of cases, where it has been implicated in multiple processes, including tumor growth, invasion of adjacent tissue, metastasis, and resistance to therapy [2, 3, 8-12].

Meta-analysis of studies of CXCR4 expression in multiple cancers including prostate cancer, non-small cell lung cancer, pancreatic ductal adenocarcinoma and others concluded that CXCR4 expression is associated with a poor prognosis and lower overall survival [13-16]. Together, these data have suggested that CXCR4 expression on cancers can serve as a correlate of aggressive biological behavior and that CXCR4 itself could be a potential therapeutic target [17]. Consequently, there has been interest in developing new imaging tools for detecting and quantifying CXCR4 on cancers in order to aid in prognostication and treatment [18, 19].

Recently, a second receptor that binds CXCL12 has been described, initially named CXCR7, and ongoing studies indicate that CXCR7 contributes to CXCR4/CXCL12 biology [20]. CXCR7 has been renamed ACKR3 (*atypical* chemokine receptor 3), because it is a seven-transmembrane domain chemokine binding protein that does not signal through heterotrimeric G proteins [20-22]. A role has been established for ACKR3 in the activities of CXCR4 and CXCL12 through ACKR3’s function as a binding protein that helps to shape CXCL12 gradients *in vivo* [23]. ACKR3 is also of interest for its possible role in cancer [24, 25].

As part of a project to develop tools for quantifying CXCR4 on cancers non-invasively in humans, we re-examined CXCR4 expression on multiple cancers by immunohistochemistry (IHC) using a well validated antibody and staining protocol. We found that CXCR4 was expressed on significant percentages of major types of kidney, lung, and pancreatic adenocarcinomas. Remarkably, we found very high expression of CXCR4 on some samples of adrenocortical carcinoma (ACC). This observation led us to focus on ACC as a model cancer in which to study the detection of CXCR4 on tumors.

ACC is a rare malignancy occurring in about 0.7-2.0 cases per million population per year, and is responsible for 0.2% of all cancer deaths in the United States [26]. Currently, the main curative treatment for ACC is surgery, with an overall 5-year survival rate for all patients undergoing tumor resection of approximately 40% [27-29]. Surgery for removal of recurrent tumor, including metastatic lesions can also prolong survival ([26] and unpublished data). Non-surgical treatment options include chemotherapy, radiotherapy and thermal ablation.

Plerixafor is a CXCR4 antagonist approved by the FDA for the mobilization of hematopoietic stem cells [25, 30]. We have previously reported the production of ^64^Cu-plerixafor [31] and studies in mice demonstrating the ability of this agent to image CXCR4-expressing tissues and cancers [31, 32]. We describe here a first-in-human study using ^64^Cu-plerixafor for PET imaging in an ACC patient undergoing resection of metastases. This study showed heterogeneity in CXCR4 expression among metastatic nodules, and good correlations among PET SUVs, CXCR4 staining, and *CXCR4* mRNA. Moreover, we found that in this patient CXCR4 expression correlated with the lesions’ rates of growth. PET imaging of CXCR4 offers a non-invasive means of assessing CXCR4 expression that could prove useful in clinical studies, including studies targeting CXCR4.

## RESULTS

### ACC expresses high levels of CXCR4

**Figure 1:**
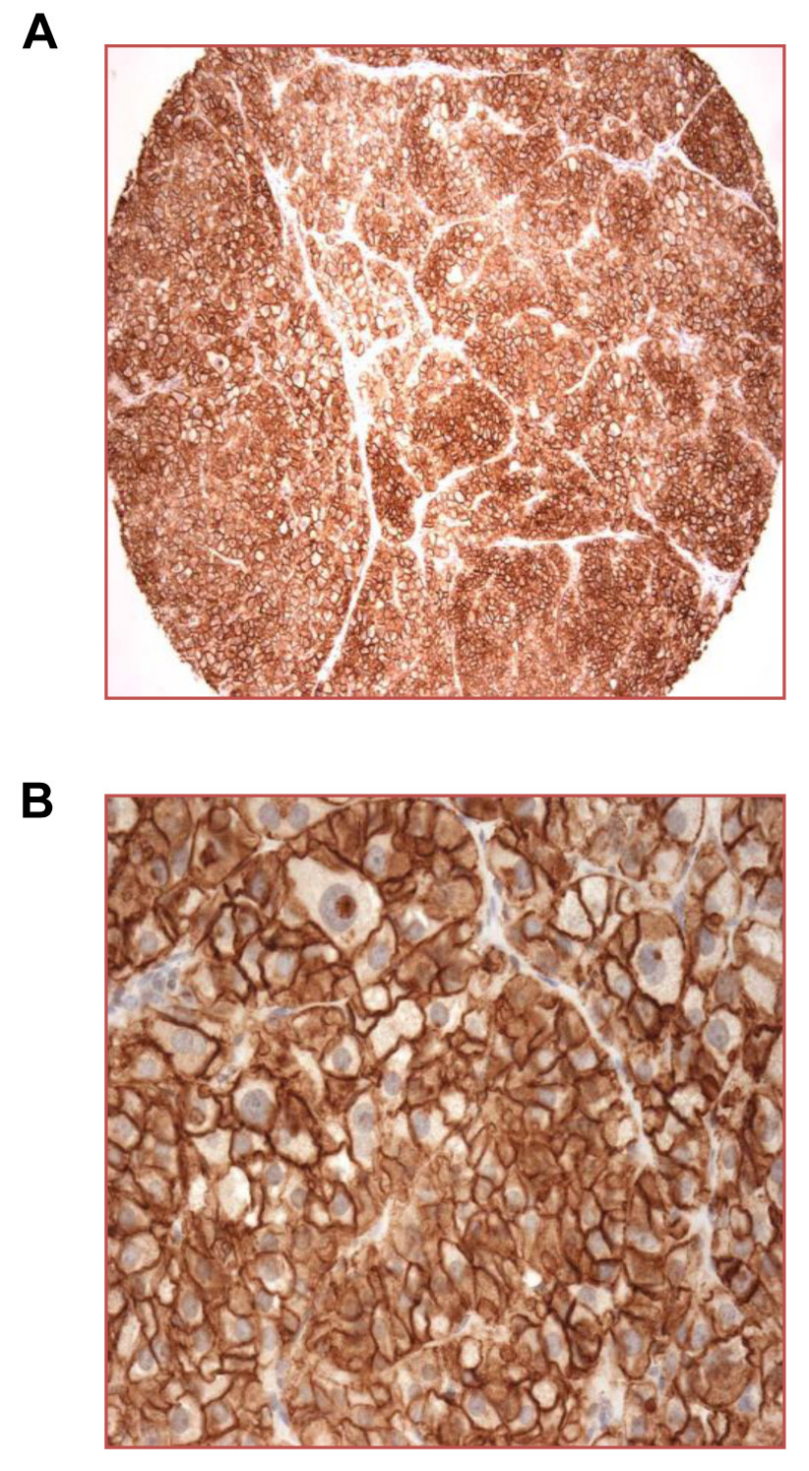
High expression of CXCR4 on cell surfaces of ACC A multi-tumor tissue array was stained for CXCR4 using IHC with visualization using DAB (3, 3’-diaminobenzidine). One sample of ACC is shown at X 100 **A.** and X 400 **B.** magnifications. An adrenal cortical adenoma on the same array showed similarly high staining for CXCR4.

Studies examining expression of CXCR4 in cancer have used multiple antibodies, with some showing predominantly cytoplasmic and nuclear staining. Nuclear localization of CXCR4 is controversial [33], and some anti-CXCR4 antibodies showing nuclear staining have been unreliable in distinguishing CXCR4^+^ versus CXCR4^-^ cells [7]. For staining tissues for CXCR4, we chose an antibody shown to stain CXCR4 on cell membranes, with no signal in nuclei or in CXCR4^-^ cells and tissues [7] (see Materials and Methods and Supplementary Figure 1). Staining a multi-cancer/multi-tumor array identified ACC expressing high levels of CXCR4 (Figure 1). We also detected expression of CXCR4 on a number of other primary and/or metastatic cancers, including those of the breast, kidney, and lung (Table 1). Based on these results and the published data on expression of CXCR4, we stained for CXCR4 on additional examples of a variety of primary and metastatic cancers. For non-ACC cancers, we used multi-case tissue arrays. Among the primary tumors for which we had a good number of cases, we found significant percentages of CXCR4^+^ cases for squamous cell carcinoma of the lung, clear cell renal cell and papillary carcinomas of the kidney, and ductal adenocarcinoma of the pancreas (Table 2). It is important to note that our expression scoring did not consider CXCR4 staining on tumor vasculature, which was often CXCR4^+^ (Supplementary Figure 1, Supplementary Figure 4, below, and data not shown).

**Table 1:** CXCR4 expression detected by IHC of tumor array

**Negative for CXCR4**^a^	**Positive for CXCR4**
Astrocytoma^b^ (1)^c^	Adrenal gland cortical adenoma (1)
Bladder transitional cell carcinoma (2)	Adrenal gland cortical carcinoma^b^ (1)
Bone chondrosarcoma (1)	Breast cancer^b^ metastatic to lymph node (1)
Bone osteosarcoma (1)	Gastric adenocarcinoma^b^ (3)
Breast adenocarcinoma^b^ (2)	Kidney clear cell carcinoma^b^ (2)
Cervix squamous cell carcinoma (2)	Lung small cell carcinoma^b^ (1)
Colon adenocarcinoma^b^ (3)	Lymphoma, anaplastic large cell (1)
Colon adenocarcinoma^b^ metastaic to liver (1)	Lymphoma, Hodgkin (Reed-Sternberg cells) (1)
Colon signet ring cell carcinoma^b^ metastatic to ovary (1)	Meningioma, malignant^b^ (1)
Esophagus squamous cell carcinoma^b^ (3)	Ovary adenocarcinoma^b^ (2)
Esophagus squamous cell carcinoma^b^ metastatic to lymph node (1)	Ovary granulosa cell tumor (1)
Gastrointestinal carcinoma metastatic to lung (1)	Skin squamous cell carcinoma (1)
Head and neck, hard palate adenocarcinoma (1)	
Head and neck, tongue squamous cell carcinoma (1)	
Head and neck, nasopharyngeal carcinoma^b^ (1)	
Liver hepatocellular carcinoma^b^ (4)	
Lung squamous cell carcinoma^b^ (2)	
Lung adenocarcinoma^b^ (1)	
Lymphoma, non-Hodgkin B-cell (1)	
Meningioma^b^ (2)	
Nasal cavity melanoma^b^ (1)	
Pancreas adenocarcinoma^b^ (1)	
Prostate adenocarcinoma^b^ (2)	
Rectum adenocarcinoma^b^ (3)	
Salivary gland adenoid cystic carcinoma (1)	
Small intestine adenocarcinoma (1)	
Testis seminoma (2)	
Thyroid adenocarcinoma^b^ (2)	
Uterus endometrial adenocarcinoma (2)	

^a^Negative tumor has < 5% of the cancer cells staining for CXCR4.

^b^Results of staining for CXCR4 in additional samples of these tumor types are presented in Tables 2-6. Data for this table are from Pantomics array MTU951, and some cases overlap with those on arrays used for Tables 2-4.

^c^Numbers of cases for each cancer type on tumor array. In the negative column, all cases were negative, whereas in the positive column, one or more of the cases of the cancers listed were positive.

**Table 2:** CXCR4 expression on selected cancers

Breast	Total samples	Positive for CXCR4	Positive for CXCR4 (%)
Ductal carcinoma in situ	4	0	0
Invasive ductal carcinoma	71	5	7
Invasive lobular carcinoma	7	0	0
**Colorectal**			
Colon adenocarcinoma	47	3	6
Colon mucinous adenocarcinoma	5	0	0
Colon squamous cell carcinoma	1	1	100
Rectum adenocarcinoma	7	0	0
**Kidney**			
Chromophobe renal cell carcinoma	6	0	0
Clear cell renal cell carcinoma	94	11	12
Granular cell and mixed granular and clear cell	11	3	27
Papillary and mixed papillary and clear cell carcinoma	14	5	36
Squamous cell carcinoma	6	3	50
Transitional cell carcinoma	21	0	0
**Lung**			
Adenocarcinoma	13	0	0
Adenosquamous carcinoma	11	1	9
Bronchioloalveolar carcinoma	11	0	0
Small cell carcinoma	3	3	100
Squamous cell carcinoma	49	9	18
Undifferentiated carcinoma	5	3	60
**Melanoma**	37	1	3
**Nervous system tumors**			
Anaplastic astrocytoma	8	2	25
Astrocytoma	29	2	7
Glioblastoma multiforme	5	0	0
Malignant meningioma	5	0	0
Meningioma	20	0	0
Neuroblastoma	3	1	33
Oligodendroglioma	5	0	0
Schwannoma	4	0	0
**Pancreas**			
Adenosquamous carcinoma	8	2	25
Ductal adenocarcinoma	126	37	29
**Prostate adenocarcinoma**	91	2	2

Overall, CXCR4 expression appeared to be greater in metastatic cancers (Tables 3 and 4), which was supported by comparisons using Fisher’s exact test. For squamous cell carcinoma of the lung and clear cell renal cell carcinoma, frequencies of CXCR4^+^ cases were significantly higher in metastatic versus primary cancers, with *P*= 0.01 and *P*=0.02, respectively. In addition, for the cases of clear cell renal cell carcinoma, the array contained matching primary and metastatic lesions. For three out of four of the CXCR4^+^ metastatic lesions, the corresponding primary tumors were also CXCR4^+^, as compared with 11 CXCR4^+^ out of 94 unselected primary carcinomas, a difference that was significant, *P*=0.009. It was also noteworthy that out of the 17 metastatic squamous cell cancers whose sites of origin included colon (1 of 17), esophagus (3), larynx (1), lung (3), nasopharynx (4), penis (1), and unknown tissues (4), 15 were CXCR4^+^ (Table 3). Most of the samples of metastatic cancers came from lymph nodes, and overall approximately 30% of cancer samples from lymph nodes were CXCR4^+^ (Table 4).

**Table 3:** CXCR4 expression on metastatic lesions, organized by primary cancers

Primary cancers	Total samples	Positive for CXCR4	Positive for CXCR4 (%)
Adenocarcinoma of unknown site	18	5	28
Breast, carcinoma	9	3	33
Colon, carcinoma	47	5	11
Colon, mucinous carcinoma	5	1	20
Colon, signet ring cell carcinoma	4	1	25
Colon, squamous carcinoma	1	1	100
Esophagus, squamous carcinoma	3	2	67
Gastric carcinoma	3	0	0
Kidney, clear cell carcinoma	7	4	57
Kidney, carcinoma, type not specified	1	1	100
Kidney, sarcomatoid carcinoma	1	0	0
Larynx, squamous carcinoma	1	1	100
Liver, hepatocellular carcinoma	2	0	0
Lung, adenocarcinoma	1	1	100
Lung, squamous cell carcinoma	3	3	100
Melanoma	12	1	8
Nasopharynx, carcinoma	4	4	100
Ovary, mucinous cystadenocarcinoma	1	0	0
Pancreas, carcinoma	1	0	0
Penis, squamous carcinoma	1	0	0
Rectum, carcinoma	7	0	0
Rectum, mucinous carcinoma	1	0	0
Squamous cell carcinoma of unknown site	4	4	100
Thyroid, follicular carcinoma	1	0	0
Thyroid, papillary carcinoma	4	1	25
All samples	142	38	27

**Table 4:** CXCR4 expression on metastatic lesions, organized by metastatic sites

Sites of metastases	Total samples	Positive for CXCR4	Positive for CXCR4 (%)
Adrenal gland	2	2	100
Bone	1	0	0
Brain	4	0	0
Greater omentum	7	1	14
Intestine	2	0	0
Liver	11	1	9
Lung	5	2	40
Lymph node	100	31	31
Ovary	3	0	0
Peritoneum	4	1	25
Spleen	2	0	0
Thyroid	1	0	0
All samples	142	38	27

**Figure 2:**
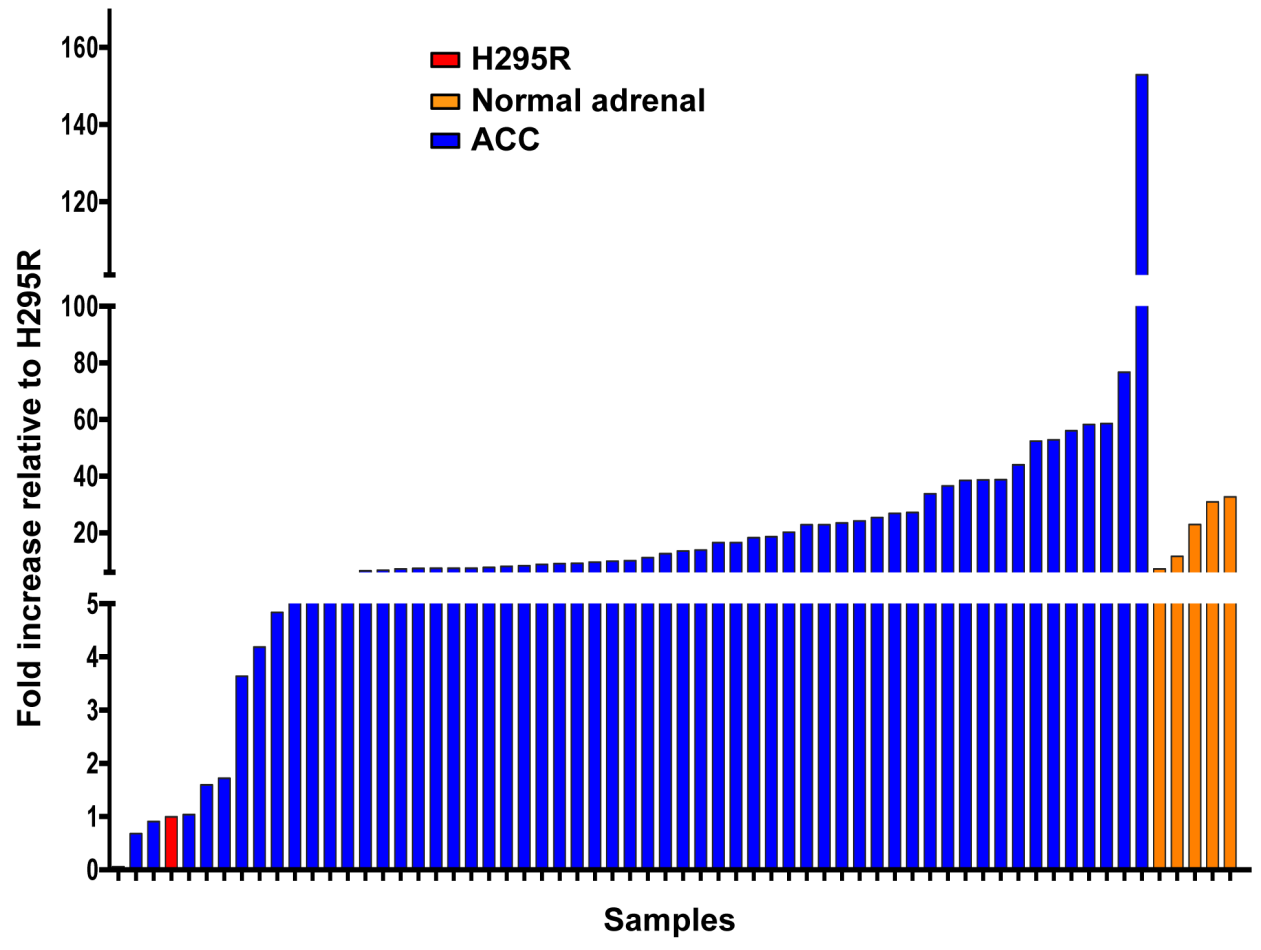
*CXCR4* is expressed in metastatic ACC Expression of *CXCR4* mRNA was determined by RT-PCR for the ACC cell line, H295R (red), five normal adrenals (orange), and 58 ACC metastases (blue) from 57 patients. After normalization to measurements of 18S rRNA, values for all samples were normalized to the value for the H295R cell line.

In order to estimate CXCR4 expression by metastatic ACC tumors more reliably, we performed additional IHC on 28 metastases from 27 patients treated at the National Institutes of Health (NIH). Seventy-five percent of the lesions were positive for CXCR4 (score > 0); 25% had scores of 12, the highest score possible (Table 5). Finally, as shown in Figure 2, we performed real-time RT-PCR analysis on 58 ACC metastases from 57 patients, 5 normal adrenals and the adrenal cancer cell line, H295. By this method, expression of *CXCR4* could be detected in all tumor samples. Levels of *CXCR4* expression extended over a wide range and exceeded the average level for the normal adrenal glands for 21 of the 58 ACC metastases.

**Table 5:** CXCR4 IHC scores of multiple ACC lesions

Age/ Sex	Score
28F	2
32M	4
33F	2
54F	1
41F	0
32F	0
51F	1
51F	4
24F	12
49F	0
64F	0
32F	12
35F	6
56M	12
52F	0
54M	12
51F	0
55M	0
45F	12
56F	9
52F	6
51M	8
53F	12
54F	4
68F	12
64M	6
72F	9
57F	8

### Expression of *CXCR4* correlates with expression of chemokine/chemokine receptor genes

In order to identify a correlation, if any, of *CXCR4* expression with that of other genes or specific biological pathways, we performed a microarray analysis using mRNA from 57 metastatic lesions removed from 42 patients (along with five normal adrenals and the H295R cell line). In our analysis, we assumed that only genes with highly variable expression (a standard deviation > 1) would be informative, thereby focusing on a set of 2,837 gene probes. The probes where then clustered into 105 groups based on Pearson similarity of expression across the tumor samples. The cluster analysis excluded the four *CXCR4* probes to remove potential bias in subsequent analysis. In order to enhance the significance of the correlations between *CXCR4* and gene networks, a pattern of a gene’s expression was considered related to *CXCR4* expression if the gene’s individual probes correlated with *CXCR4* expression, and the gene was also a member of a cluster whose centroid correlated with *CXCR4* expression.

*CXCR4* correlations for all probes are plotted in Supplementary Figure 2, the Y-axis indicating the individual probe-probe correlation with *CXCR4*, the X-axis the correlation with the probe cluster’s centroid with *CXCR4*. There were 403 gene probes appearing in clusters with high correlation with *CXCR4* that also individually showed high correlation with *CXCR4* expression (both with Pearson’ s coefficient > 0.2). Gene Ontology term enrichment analysis of these 403 probes showed associations with immunological processes, and with the chemokine system and chemotaxis (Supplementary Figure 3).

Remarkably, the list of genes correlating with *CXCR4* expression included twelve chemokine and two chemokine receptor genes (Supplementary Table 1).

As a control, we analyzed the collection of 191 gene probes within clusters showing negative correlations with *CXCR4* that individually also showed large negative correlations with *CXCR4* expression (both with Pearson’s coefficient < - 0.2, Supplementary Figure 2). Gene Ontology term enrichment analysis of these genes found no associations with immunological processes or with the chemokine system (Supplementary Figure 4), and the list of genes did not include chemokines or chemokine receptors (data not shown).

*CXCL12*, the gene encoding the CXCL12 chemokine, was not among the genes whose expression correlated with *CXCR4*. Additionally, staining of 15 of the ACC cases for CXCL12 by IHC, 10 of which were CXCR4^+^ by IHC (data not shown), failed to reveal CXCL12 in cancer cells. Nonetheless, expression of *CXCL12* could be readily detected in the ACC metastases on the microarrays (see GEO Series accession number GSE90713 per Materials and Methods), and CXCL12 was identified by IHC in some tumor vessels and adjacent normal tissues (Supplementary Figure 5).

**Figure 3:**
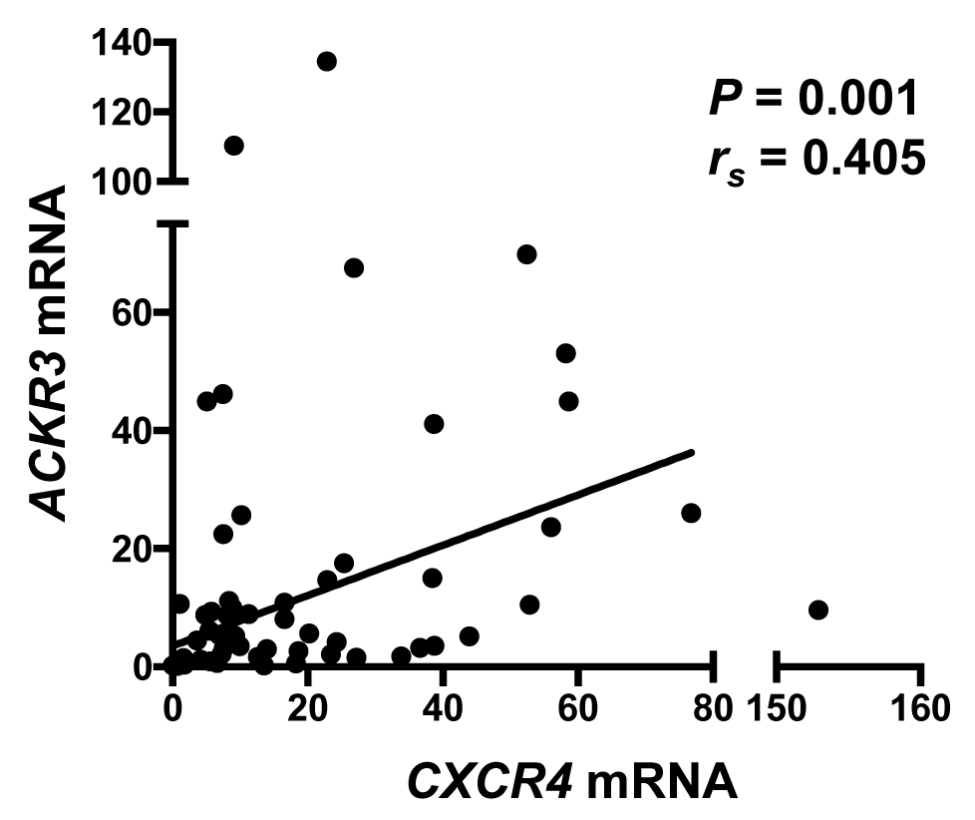
Expression of *CXCR4* and *ACKR3* are positively correlated in metastatic ACC Expression of *ACKR3* mRNA was determined by RT-PCR for the 58 ACC metastases analyzed in Figure 2 and, after normalization to measurements of 18S rRNA and to the value for the H295R cell line, compared with expression of *CXCR4*. From a Spearman analysis, the rank correlation coefficient (*r_s_*) and *P* were calculated.

Of particular interest, one chemokine receptor gene associated with expression of *CXCR4* was *ACKR3*, the gene encoding the other receptor known to bind CXCL12. We confirmed this observation by demonstrating a significant correlation between *CXCR4* and *ACKR3* expression in 57 ACC metastatic lesions by real-time RT-PCR (Figure 3).

### Semi-quantitative, non-invasive determination of CXCR4 expression on metastases of ACC using ^64^Cu-plerixafor

Determining expression of CXCR4 in individual tumors would be valuable for investigating the significance of CXCR4 in ACC and other cancers, and for selecting patients for studies using CXCR4-targeting therapies. This suggested a potential advantage to the noninvasive assessment of CXCR4 expression in cancer. We, along with others, have previously shown in mouse models that CXCR4 can be evaluated *in vivo* using the PET tracer ^64^Cu-plerixafor (also known as ^64^Cu-AMD3100) [31, 32, 34]. We extended these studies to a patient with metastatic ACC under a clinical protocol (ClinicalTrials.gov identifier NCT02069080) that required that initial subjects have pre-existing biopsies that stained positive for CXCR4 by IHC.

**Figure 4:**
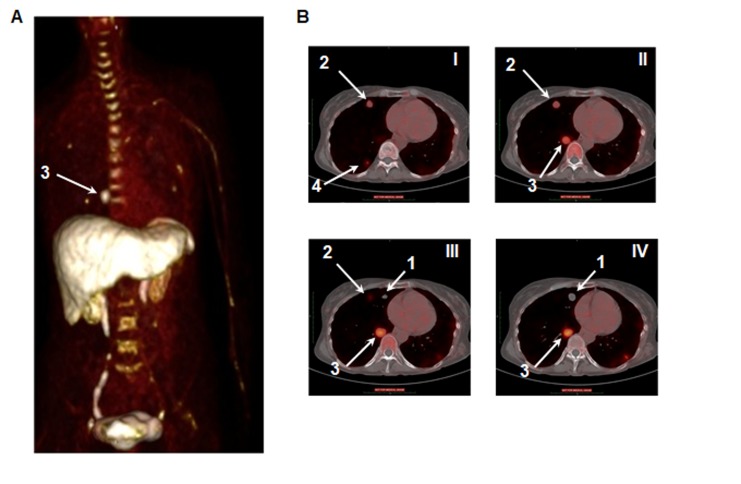
^64^Cu-plerixafor identifies pulmonary metastases of ACC **A.** PET Maximum Intensity Projection (MIP) of a patient with metastatic ACC 40 minutes following injection of ^64^Cu-plerixafor. **B.** Thoracic transaxial plane images of PET as in A with CT co-registration. Images are rostral (I) to caudal (IV). Right lung nodules, as indicated by the white arrows, were numbered prior to resection to allow for subsequent analyses. Nodule 1 was the only target nodule without focal radiotracer uptake.

The study subject was a 57-year old woman who underwent pulmonary metastasectomies as part of her medical care under a separate NIH protocol. Following a first surgery that removed nodules in the left lung, IHC of one nodule revealed CXCR4 expression with a score of 8 out of a maximum of 12 (data not shown and see Materials and Methods). PET/CT scanning using ^64^Cu-plerixafor was performed six and five days prior to a second surgery for removal of nodules in the right lung. Per protocol, a dose of 8.6 mCi of ^64^Cu-plerixafor (specific activity of 8.76 mCi/μg plerixafor) was administered followed immediately by three consecutive scans and two additional scans at approximately 4 and 24 hours post injection. A representative projection of a PET image is shown in Figure 4A.

Similar to results in mice, the liver had the highest uptake of the tracer, with unbound tracer excreted through the kidneys [31, 32]. Significant uptake was also seen in organs of the immune system, including spleen, vertebral bodies (bone marrow), and lymph nodes (Figure 4 and Supplementary Figure 6). Of additional interest, uptake of ^64^Cu-plerixafor was absent from a number of vertebral bodies in the thoracolumbar spine that were within the region of prior radiation therapy (Figure 4 and Supplementary Figure 6). Dosimetry for ^64^Cu-plerixafor calculated from this single patient was 0.204 rem/mCi, and a total of 1.75 rem from the dose of 8.6 mCi. The organs that contributed the most for exposure were the liver and kidneys (0.0638 and 0.00243 rem/mCi, respectively). PET/CT sections (Figure 4B) showed variable uptake in the multiple pulmonary nodules.

**Figure 5:**
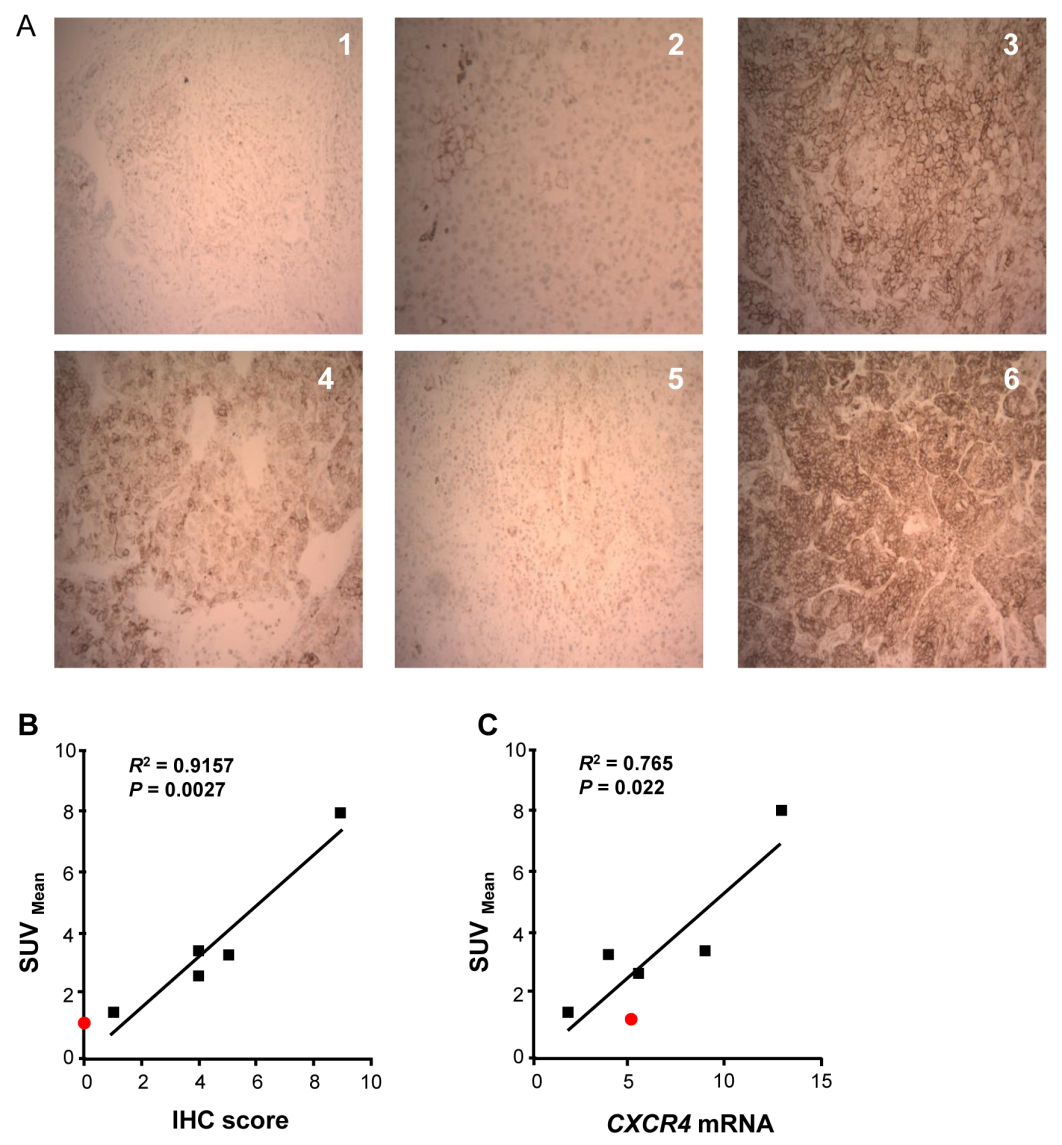
Uptake of ^64^Cu-plerixafor correlates with expression of CXCR4 **A.** IHC staining of sections from the six resected pulmonary nodules. Nodule 1 was a chondroma and nodules 2-6 were metastatic ACC. Magnification is X 100. **B.** Linear regression analysis of CXCR4 IHC score vs. ^64^Cu-plerixafor SUV_mean_ for the five excised ACC nodules. **C.** Linear regression analysis of *CXCR4* mRNA vs. ^64^Cu-plerixafor SUV_mean_ for the five excised ACC nodules. After normalization to measurements of 18S rRNA, values for *CXCR4* mRNA were normalized to the value for the H295R cell line as in Figure 2. Red symbol in B and C corresponds to nodule 1 (chondroma), which was not included in statistical analyses. *R*^2^ is the coefficient of determination.

CXCR4 staining of the six excised nodules using IHC, as shown in Figure 5A, revealed a range of CXCR4 expression, with virtually no CXCR4 staining in nodule #1, which pathologic analysis showed to be a chondroma rather than ACC. The standardized uptake values (SUV) at three hours after injection of the ^64^Cu-plerixafor PET tracer were compared to both CXCR4 IHC scores and *CXCR4* mRNA levels in samples from the five excised ACC nodules. The SUV’s showed significant correlations with both the IHC scores (*P*= 0.0027, Figure 5B) and mRNA levels (*P* = 0.022, Figure 5C), demonstrating that uptake of ^64^Cu-plerixafor as determined by PET can be used to quantify expression of CXCR4.

Beyond the heterogeneity found in CXCR4 expression among ACC metastases among different individuals as shown in Figure 2 and Table 5, the finding of significant nodule-to-nodule heterogeneity in CXCR4 expression among the lesions present at one time in this single patient led us to analyze CXCR4 expression by immunostaining in multiple metastases of ACC resected at various times from four additional patients. As shown in Table 6, heterogeneity in CXCR4 expression was also found among pulmonary nodules resected from some other patients, including in patient 3, where a total of ten nodules removed in Years 1-3 were all CXCR4^+^, whereas the five nodules removed in Year 4 scored as 1 (one nodule) or 0 (four nodules), suggesting a change in CXCR4 expression in this patient’s metastases over time. The scores in Years 2, 3, and 4 were significantly different by one-way ANOVA (*P*=0.0005), as were pairwise comparisons using Student’s t-test between Year 4 and Year 2 or Year 3, *P*=0.0017 and *P*=0.0002, respectively.

**Table 6:** CXCR4 IHC scores of lesions from individual patients over time

	Year of resection^a^	Score
Patient 1	Year 1^b^	12
	Year 2	12
	Year 3	12
	Year 3	12
Patient 2	Year 1	0.5
	Year 2	0
	Year 2	0
	Year 2	6
	Year 2	1
	Year 3	6
	Year 3	6
	Year 3	12
	Year 3	3
	Year 5	6
	Year 5	4
	Year 9	0
Patient 3	Year 1	2
	Year 2	6
	Year 2	12
	Year 2	6
	Year 2	4
	Year 2	6
	Year 3	6
	Year 3	12
	Year 3	8
	Year 3	8
	Year 4	1
	Year 4	0
	Year 4	0
	Year 4	0
	Year 4	0
Patient 4	Year 1	0
	Year 2	0
	Year 3	0

^a^For each patient, the earliest year in which the scored lesions were resected is designated Year 1.

^b^Each row corresponds to a single, separate metastatic nodule.

### Expression of CXCR4 correlates with growth of ACC nodules

**Figure 6:**
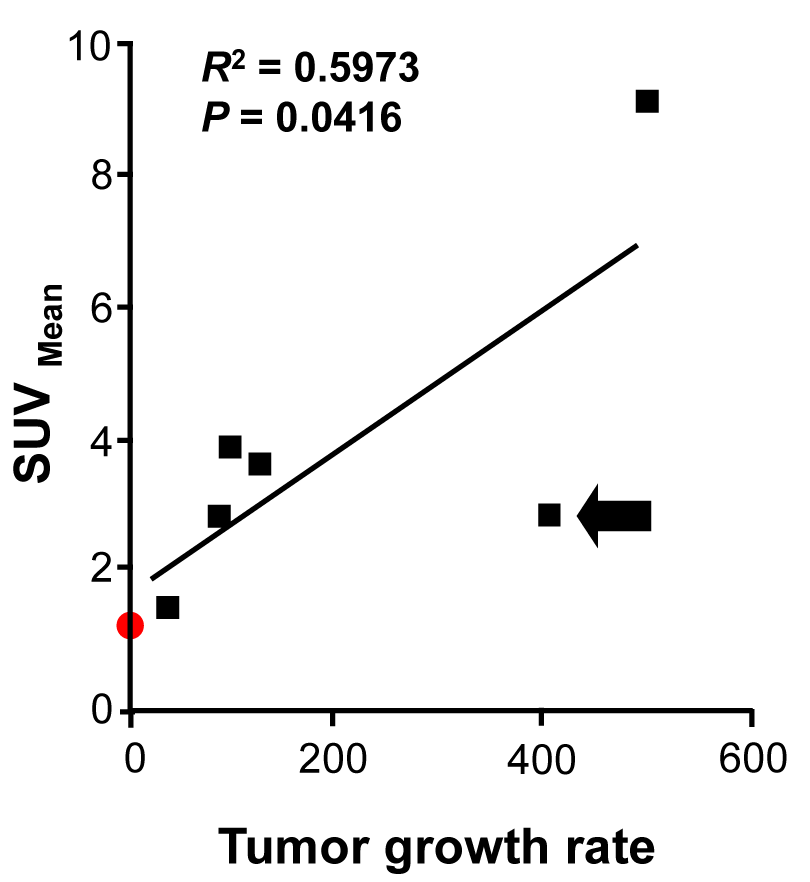
^64^Cu-plerixafor SUV_mean_ correlates with growth rate for ACC metastases Linear regression analysis is shown for ^64^Cu-plerixafor SUV_mean_ vs. tumor growth rate (see Materials and Methods) for six pulmonary nodules over the four months before ^64^Cu-plerixafor PET/CT. Arrow indicates a nodule of presumed ACC that was not available for analysis in Figure 5. Red symbol corresponds to nodule 1 (chondroma), which was not included in the statistical analysis. *R*^2^ is the coefficient of determination.

Our quantification of differences in CXCR4 expression in multiple tumor nodules in a single patient at one time offered a good opportunity to assess potential biological correlates of CXCR4 expression. Using available high-resolution lung CT scans obtained 4 months and just before surgical resection, the growth rates of the patient’s individual nodules were calculated based on the lesions’ volumes [35]. We found a significant correlation (*P* = 0.0416) between tracer SUV and tumor growth rates (Figure 6).

## DISCUSSION

By screening cancer tissue arrays by IHC, we discovered that ACC expresses high levels of CXCR4, and extended these findings using IHC and gene array analyses. We demonstrated that in ACC metastases expression of *CXCR4* correlates with expression of other chemokine/chemokine receptor genes including *ACKR3*, the gene encoding the other receptor known to bind CXCL12. Using ^64^Cu-plerixafor, we were able to determine CXCR4 expression in ACC metastases non-invasively, and demonstrate significant correlations of the SUVs and expression of CXCR4 protein and mRNA as measured by IHC and RT-PCR, respectively, in five pulmonary metastases. Moreover, in this same patient we found that expression of CXCR4 as determined by ^64^Cu-plerixafor PET correlated with growth of the metastatic ACC nodules.

We also performed IHC studies of non-ACC cancers. Staining of primary cancers revealed significant frequencies of CXCR4^+^ samples in cancers of the kidney, lung, and pancreas. Nonetheless, these frequencies were generally lower than reported in published data [3]. Moreover, for other cancers where high frequencies of CXCR4 staining have been reported, such as primary tumors of the breast, colon, and prostate [3], we detected CXCR4 in fewer than 10% of cases. There are several possible reasons for our comparatively low rates of CXCR4 positivity. A limitation of our studies was the use of tissue arrays, which contain small tissue cores. Given the heterogeneity in expression of CXCR4 found routinely within individual tumor samples (as we found, for example, in ACC metastases), depending on a number of factors such as scoring criteria, our array data may have contained a higher frequency of “negative” scores as compared to surveys using significantly larger tissue sections for individual cancers. In addition, every antibody/staining protocol combination will have its own limit of detection. Our data for a large number of ACC metastases, for example, showed that although all metastases contained detectable *CXCR4* mRNA, CXCR4 could not be detected by IHC in approximately 25% of samples examined. Nonetheless, it is also important to note that we used an antibody whose specificity in IHC has been well validated by others, as well as in our own hands, whereas many of the studies in the literature have used antibodies that have been poorly characterized for this application, and often produce nuclear staining, whose significance is unclear [7]. Taken together, these considerations suggest that our IHC studies are likely to be highly specific but not highly sensitive, detecting tumors with substantial expression of CXCR4 and thereby providing a reliable lower-limit estimate of the frequencies of CXCR4-expressing tumors.

From the initial studies of CXCR4 in cancer [36] there has been a focus on the role of the receptor in metastasis [2]. It is of interest, therefore, that we found higher frequencies of CXCR4^+^ samples from metastatic as compared with primary tumors for squamous cell cancer of the lung and clear cell renal cell cancer. Particularly striking were our findings for metastatic squamous cell cancers originating from a range of tissues, for which 15 of 17 cases were CXCR4^+^. The majority of metastatic samples on the arrays were obtained from lymph nodes, and approximately 30% of the metastases in lymph nodes were CXCR4^+^. We can only speculate as to the reason(s) for the increased frequencies of CXCR4^+^ samples in metastatic versus primary tumors for some (but not all) of the cancers. The comparatively high frequencies for some cancers did not bear a simple relationship to the site of metastasis, since most metastases were to lymph nodes for both the metastases that were CXCR4^+^ and those that were CXCR4^-^. For renal cell clear cell carcinoma, where we had matched primary and metastatic samples, our data are consistent with preferential metastasis by CXCR4^+^ primary cancers as a basis for the enrichment of CXCR4^+^ cases among the metastases, which is in line with some published findings [37].

Cluster analysis of the gene array data including ACC samples from 42 patients revealed a significant correlation of *CXCR4* expression with other genes in the chemokine system, including twelve chemokines and two chemokine receptors. We are not aware of previous data showing the correlation of expression of *CXCR4* with many other genes within the chemokine system in human tumors. In considering these results, however, it is worth remembering that the gene arrays and RT-PCR data do not allow us to identify the cell types within the tumor samples expressing the relevant mRNAs and proteins.

*CXCL12*, which encodes the ligand for CXCR4, was not among the chemokine genes we identified whose expression correlated with expression of *CXCR4* in the ACC metastases, nor did we find CXCL12 in the cancer cells by IHC. Nonetheless, the data do not suggest that CXCL12 was unavailable for activating CXCR4 on the cancer cells. The microarray data detected expression of *CXCL12* mRNA in the ACC samples, and CXCL12 may have been made by cancer cells below the limit of detection by IHC. In any case, CXCL12 can be found in serum, in tumor capillaries, and in normal tissue at sites of metastasis, including lung and liver as shown in our IHC and as reported previously [36].

The chemokines encoded by the genes that we did find to be correlated with *CXCR4* expression are ligands for nine chemokine receptors found on many types of leukocytes, including neutrophils, monocytes/macrophages, dendritic cells, T cells, B cells, NK cells, and innate lymphoid cells - and have been reported to have a range of activities in cancers [38]. Among many examples of these chemokines’ activities, CXCL2 and CCL2 have been shown to recruit myeloid-derived suppressor cells (MDSC) [39, 40]; CCL2 and CCL5 have been implicated in recruitment of tumor-supporting macrophages [41-43]; CCL5 has been shown to have direct pro-tumorigenic activity in metastatic colon cancer [44]; CCL18 induces epithelial-mesenchymal transition in breast cancer [45]; and CCL20 recruits IL-22-producing CD4^+^ T cells that contribute to tumorigenesis in colon cancer [46]. One possible basis for co-expression of these genes might be the activity of NF-κB, which has been reported to induce expression of many of these chemokines, as well as the three receptors whose mRNAs we detected, CXCR4, CXCR7, and CX3CR1 [47-50].

Co-expression of *CXCR7/ACKR3* with *CXCR4* was confirmed using real-time RT-PCR and is of particular interest given that CXCR4 and ACKR3 cooperate functionally through their distinct modes of interacting with their shared chemokine, CXCL12. Both the *CXCR4* and *ACKR3* genes can be induced by hypoxia, and by the transcription factor GLI1 (as well as NF-κB) [48, 49, 51, 52], suggesting possible mechanisms underlying co-expression. It is of interest that expression of *CXCR4* and *ACKR3*, which direct the collective migration of cells in the developing zebrafish, is regulated in part by Wnt/β-catenin [53], which has also been suggested to contribute to ACC [54]. ACKR3 can act by signaling independently through β-arrestin-mediated pathways [55], and/or by directly affecting CXCR4 signaling through the formation of ACKR3/CXCR4 heterodimers [56, 57], and/or by sequestering CXCL12 and thereby regulating the concentrations of chemokine and shaping the chemokine gradients to which CXCR4-expressing cells respond [21, 23, 58, 59].

Co-expression of CXCR4 and ACKR3 has been reported both on separate cells and on the same cells within primary cancers [60]. It has been suggested that ACKR3 and CXCR4 cooperate in enhancing tumor growth [60, 61], sometimes through separate effects on cancer cells and angiogenesis [62], and blocking ACKR3 has been shown to diminish growth of tumors in mouse models [20]. Like CXCR4, ACKR3 has been considered a potential target for therapy of a number of cancers, including glioblastoma, endometrial carcinoma, and lung cancer [63-65].

The other chemokine receptor gene whose expression correlated with that of *CXCR4* was *CX3CR1*, which is expressed in NK cells, subsets of monocytes, CD4^+^ and CD8^+^ T cells [22], as well as prostate cancer [66] and various experimental cancers and cancer cell lines [67-70]. Again, although the gene array data do not allow us to identify which cell type(s) in the ACC tumors were expressing *CX3CR1*, CX3CR1 has some similarities of interest with CXCR4 related to potential roles on malignant cells. Just as for CXCR4, CX3CR1 is up-regulated by the hypoxia-responsive transcription factor, HIF-1*α* [68]. In addition, CX3CR1 has been implicated in metastasis of prostate cancer cells [66] and breast cancer cells [71] to bone, and CXCR4 may have a particular role in bone metastasis [8, 72]. Bone is a known site for metastasis of ACC [29, 73], so that CX3CR1 and CXCR4 may contribute cooperatively to this proclivity. Taken together, the correlations of expression of CXCR4 with these chemokines and these other chemokine receptors suggest the potential for cooperative activities of these proteins within ACC metastases, likely through direct effects on cancer cells and tumor-associated endothelial cells, as well as through effects on leukocytes in the tumor microenvironment.

The heterogeneity of CXCR4 expression found within various types of cancers, including ACC, suggests that a method to detect and quantify CXCR4 on tumors non-invasively would be valuable in investigating CXCR4 in cancer biology, and in selecting and following patients in studies targeting CXCR4 as anti-cancer therapy. We showed previously that ^64^Cu-plerixafor could be used to image CXCR4 in mouse tissues [31], and we and others also showed that ^64^Cu-plerixafor could be used to visualize CXCR4 expression on experimental tumors [32, 34]. We show here the first data for ^64^Cu-plerixafor as a probe to detect CXCR4 expression by PET/CT in a patient. We detected significant uptake of ^64^Cu-plerixafor in organs of the hematopoietic and immune systems, which contain high numbers of CXCR4-expressing cells, including bone marrow, lymph nodes, and spleen. A portion of the thoracolumbar spine that had been exposed to radiotherapy, presumably leading to ablation of bone marrow, showed no uptake of the probe. As in our mouse studies [31, 32], we also detected accumulation of ^64^Cu-plerixafor in the liver. Although we had found that uptake by the liver in mice was plerixafor-specific [31], low expression of CXCR4 in the liver [74, 75] makes it unlikely that this uptake is CXCR4-specific. For the pulmonary metastases of ACC, we found that the nodules’ SUVs correlated well with CXCR4 scoring by IHC and levels of *CXCR4* mRNA, suggesting that ^64^Cu-plerixafor can be used to quantify CXCR4 expression on tumors. Our results using IHC and/or ^64^Cu-plerixafor PET demonstrated significant differences in CXCR4 expression among ACC metastases within individual patients. We found differences in multiple nodules present at a single point in time, and a suggestion that metastases resected at different times from a given patient could differ in their overall levels of CXCR4 expression. Our findings emphasize the potential usefulness of a method for detecting CXCR4 expression on individual tumors that is non-invasive and avoids sampling bias.

In fact, there has been a great deal of recent interest in developing PET agents for visualizing CXCR4 expression on cancers [19]. One study used ^68^Ga-NOTA-NFB, a derivative of the peptide antagonist of CXCR4, T140, to image gliomas [76]. The most extensive published data have been with a newly developed synthetic pentapeptide labeled with ^68^Ga, ^68^Ga-pentixafor [77], which has been evaluated recently in patients with lymphoma [78], multiple myeloma [79], small cell lung cancer [80], glioblastoma [81], neuroendocrine tumors [82], and other cancers [83]. When tissue samples were evaluated, poor correlations were reported for glioblastoma and small cell lung cancer between ^68^Ga-pentixafor SUVs and CXCR4 immunoreactivity [80, 81]. The most recent data for gastro-entero-hepatic neuroendocrine tumors showed a better but still imperfect correspondence between CXCR4 positivity of tumor biopsies and ^68^Ga-pentixafor positivity by PET [82]. Uptake of ^68^Ga-pentixafor was also reported very recently for metastases of ACC, although in that study no tissue samples were available for comparing SUVs with CXCR4 expression assessed by independent methods [84]. As far as we are aware, our data include the first demonstration of a PET agent for CXCR4 for which SUVs faithfully report relative levels of expression of cell surface CXCR4 on, and *CXCR4* gene expression in, human tumors.

ACC is a rare disease for which the current best treatment with the possibility of cure is surgical resection of the primary tumor. As for most solid malignancies, new therapies are needed for treatment of non-resectable and metastatic disease. A number of studies are underway assessing the safety and efficacy of CXCR4 antagonists in patients with solid tumors, such as glioblastoma multiforme, and cancers of the pancreas, ovaries, and colon (ClinicalTrials.gov identifiers NCT02179970, NCT02737072, NCT02765165). Despite the rarity of ACC, given the high levels of expression in a majority of tumors, ACC might be considered an appropriate malignancy in which to test therapies targeting CXCR4.

## MATERIALS AND METHODS

### Human samples and clinical protocols

Human ACC samples were acquired and analyzed from study subjects after obtaining informed consent under National Cancer Institute Center for Cancer Research (NCICCR) protocol “Natural History and Tissue Acquisition Study of Adrenocortical Carcinoma”, number 14-C-0029, ClinicalTrials.gov Identifier NCT02015026, and/or under NCICCR protocols 04-C-0011, 08-C-0176, 10-C-0203, 06-C-0014, and 01-C-0129. PET/CT imaging with ^64^Cu-plerixafor was done under protocol “Imaging CXCR4 Expression in Subjects With Cancer Using ^64^Cu-Plerixafor”, number 14-I-0050, ClinicalTrials.gov Identifier NCT02069080. Protocols were approved by the Institutional Review Board of the National Cancer Institute Center for Cancer Research.

### Tumors in mice

Athymic (nude) mice were purchased from Taconic Biosciences (Hudson, NY). Mice were housed in pathogen-free conditions and experiments were performed under protocols approved by the NIH Institutional Animal Care and Use Committee. Chinese hamster ovary (CHO) cells, 3LL Lewis lung carcinoma cells, and derivatives of these cell lines expressing human CXCR4, designated CHO-XR4 and 3LL-XR4, respectively, were obtained, cultured, and injected subcutaneously into nude mice for producing local tumors as described [32]. Tumors were excised, formalin-fixed and paraffin-embedded for analysis by IHC as described below.

### ^64^Cu-plerixafor PET imaging

^64^Cu-plerixafor was synthesized according to the method as described [31] with detailed procedures according to U.S. FDA IND 107188. Briefly, 2 µg of plerixafor (Mozobil^®^, Sanofi, Gentilly, FR) in 0.4 M ammonium acetate (Sigma-Aldrich, St. Louis, MO), pH 5.5 was reacted with 20 mCi of ^64^Cu-acetate (supplied initially as ^64^CuCl_2_ by Cyclotron Facility, PET Department, Clinical Center, NIH). This mixture was vortexed and incubated at 37 °C for ∼ 1 to 1.5 hours and the labeling yield of ^64^Cu-plerixafor analyzed by instant thin layer chromatography. Thereafter the ^64^Cu-plerixafor was combined with sterile saline, passed through sterile 0.22 μm filters (Millipore, Billerica, MA), and determined to contain < 3.46 EU of endotoxin per dose (Endosafe^®^, Charles River Laboratories, Wilmington, MA) before being released for use.

The patient was injected i.v. with 8.6 mCi of ^64^Cu-plerixafor (0.98 μg plerixafor, specific activity 8.76 mCi/μg) and underwent one low dose CT scan and three PET scans during the first hour. Two additional PET/CT scans were carried out 3 and 23 hours after tracer injection. The patient was imaged on a Philips Gemini TF PET/CT (Philips Health Care, Cleveland, OH). The low-dose, non-contrast CT transmission scans (120 kVp, 60 mAs) were used for attenuation correction and co-registration.

The attenuation-corrected PET images in transaxial, coronal, and sagittal projection planes were evaluated visually, co-registered with the CT, and images were displayed using MIM Encore 5.2 (MIM Software, Inc., Cleveland, OH). Volumes of interest (VOI) containing entire lesions were drawn and SUV mean values were calculated. Dosimetry was calculated using Organ Level INternal Dose Assessment (OLINDA) Code (Version 1.1, Vanderbilt University, 2007).

### Immunohistochemistry

Tumor arrays and suppliers were as follows: BRC1021, KIC1501, KIC1502, LUC1021, NST1021, MET181, MET961, PRC1021, and MTU951 were purchased from Pantomics (Richmond, CA); CO702a, CO992, HPan-Ade150CS, KD951a, OD-CT-DgPan03-001, and PR633 were purchased from US Biomax (Rockville, MD); CK2 was purchased from Super Bio Chips (Seoul, Korea). IHC was performed similarly on tumor arrays, other human tissue samples, and tumors from mice. Paraffin-embedded sections were baked for 30 min at 55 °C and deparaffinized in xylene x 3. Samples were rehydrated by incubation in a descending gradient of alcohol and thereafter antigen retrieval was performed using low pH antigen retrieval solution (Dako, Carpinteria, CA) at 95 °C for 20 minutes or using a pressure cooker in a microwave oven for 20 minutes. Slides were blocked with 4% rat and mouse sera or Tris-buffer with 5% goat immunoglobulin , and stained with rabbit anti-CXCR4 antibody UMB-2 (Abcam, Cambridge, MA) at a dilution of 1:500 for 1 hour at room temperature. Anti-rabbit Envision + DAB kit (Dako, Carpinteria, CA) was used to visualize the binding of the primary antibody as instructed by the manufacturer. Sections from human tonsils were used as postive controls. In Supplementary Figure 1 and data not shown, we validated UMB-2, and optimized staining procedures using formalin-fixed, paraffin-embedded CXCR4^-^ and CXCR4^+^ cell lines, processed either as pellets or from tumors grown in mice, and also using sections of human tonsils. The tumors displayed in Supplementary Figure 1 had been analyzed previously using micro-PET and by flow cytometry of single-cell suspensions, demonstrating high and low levels of CXCR4 expression in the *CXCR4*-transfected CHO and Lewis lung carcinoma tumors, respectively [32]. Some photomicrographs were taken using an Olympus Bx41 microscope with objectives Plan 2x/0.05, UPlanFI 10x/0.30, UPlanFI 20x/0.50, and UPlanFI 40x/0.75, with an adaptor U-TV0.5xC using digital camera Q-imaging Micropublisher 5.0 RTV. The images were captured using “Q-Capture Version 3.1” and imported to Adobe Photoshop 7.0. Other images were obtained using an Olympus BH-2 microscope with objective SPlan 10x/0.30 and equipped with Q-imaging 12 bit camera and acquisition software.

Tissue cores on the arrays were scored as positive or negative for CXCR4 expression by the cancer cells, and all tumor arrays were scored by a single pathologist. For the ACC samples, a standard scoring system [85] was modified for the evaluation of CXCR4 expression. The H-score was defined as: (percentage quintile x average intensity of staining). The percentage quintiles were defined as follows: 0%-5% of cell staining positive, a score of 0; 6%-25% of cells staining positive, a score of 1; 26%-50% of cells staining positive, a score of 2; 51%-75% of cells staining positive, a score of 3; 76%-100% of cells staining positive, a score of 4. The average intensity of staining scale was defined as follows: weak=1, moderate=2, and strong=3. Consequently, the H-scores ranged from 0 to12 (quintile 4 x intensity of 3). For analyzing samples resected from the patient following studies using ^64^Cu-plerixafor, investigators were blinded as to the lesions’ SUV’s when scoring IHC.

### Analysis of tumor growth rates

Tumor growth rates were calculated as described [35]. Calculations were performed using an online calculator found at http://ec2-54-218-32-173.us-west-2.compute.amazonaws.com:3838/tgrShiny/. The volume of each lesion was measured using PACS software from two surgical CT scans done at one week before and four months before PET scanning using ^64^Cu-plerixafor.

### RNA isolation and real time RT-PCR

RNA was isolated using the Qiagen miRNeasy Kit (Qiagen, Valencia, CA) or PureLink RNA KIT (Ambion, Austin, TX) following the manufacturers’ instructions. In addition to tissue samples, for purposes of comparison and normalization, RNA was prepared from the H295R adrenocortical carcinoma cell line, obtained from the American Type Culture Collection, Manassas, VA. Real time RT-PCR was carried out using gene specific TaqMan probes and primers for *CXCR4* (Applied Biosystems, Foster City, CA, catalogue number Hs00607978_s1), *CXCR7/ACKR3* (catalogue number Hs00604567_m1), and 18S rRNA (catalogue number Hs99999901_s1) on an ABI StepOne instrument, and data were analyzed using SDS software (Applied Biosystems), after which values for *CXCR4* and *CXCR7/ACKR3* were normalized to values for 18S rRNA for each sample.

### Microarray data processing and analysis

RNAs from 63 samples (57 tumor samples, 5 normal adrenal tissue samples, the latter from CHTN Southern Division, Birmingham, AL) and a sample from the H295R cell line were processed on Human PrimeView arrays (Affymetrix, Santa Clara, CA). Array design (PrimeView.cdf, rev01) and genomic annotation (PrimeView.na35.annot.csv) were downloaded from Affymetrix product support (http://www.affymetrix.com/support/technical/byproduct.affx?product=primeview). Signal data were extracted from CEL files with the Affymetrix Import Engine of JMP Genomics software v. 7.0 (SAS Institute, Cary, NC) using RMA background correction, transformation to log2 after adding 16 (shifting factor), and summarization by median polish. Quantile normalization was applied across all probe intensities on all arrays. The data have been deposited in NCBI’s Gene Expression Omnibus and are accessible through GEO Series accession number GSE90713 (http://www.ncbi.nlm.nih.gov/geo/query/acc.cgi?acc=GSE90713).

### Cluster analysis

k-means clustering of probe sets was done on the basis of differences among tissue samples in the 63-sample set. In order to do this, the quantile-normalized dataset described above was further standardized for each probe set across the samples by subtracting the mean (over the tissue samples) and dividing by the standard deviation (over the tissue samples). Thus, the Euclidean distance between probe set patterns was equivalent to the inverse of the Pearson correlation. Only those probe sets with a standard deviation > 1.0 were retained for cluster analysis. The cubic clustering criterion was used to determine the optimal number of clusters. Each cluster centroid (vector of tissue sample expression values) was calculated by averaging the tissue sample values for all probe sets in the cluster. These calculations were done using JMP Genomics software (SAS Institute, Cary, NC).

### Gene ontology term enrichment analysis

Pathway analysis was carried out using Gene Ontology enRIchment anaLysis and visuaLizAtion tool (GOrilla, http://cbl-gorilla.cs.technion.ac.il ) using genes from the cluster analysis done after excluding *CXCR4* probes, for two groups of genes: one group included genes that had > 0.2 correlation with *CXCR4* probes from clusters that had mean correlations of > 0.2 with *CXCR4* probes; the second group included genes that had < -0.2 correlation with *CXCR4* probes from clusters that had mean correlations of < -0.2 with *CXCR4* probes. GOrilla results were visualized using REVIGO (http://revigo.irb.hr) [86].

### Statistical analysis

Fisher’s exacts tests were two-tailed and performed using GraphPad QuickCalcs at https://graphpad.com/quickcalcs/contingency1.cfm (GraphPad Software, La Jolla, CA). Determinations of Pearson’s and/or Spearman’s correlation coefficients, one-way ANOVA and Student’s t-tests were done using GraphPad Prism. For each correlation *R*^*2*^ or *r*_*s*_ and *P* values are shown.

## SUPPLEMENTARY MATERIALS FIGURES AND TABLE



## Abbreviations

ACC: adrenocortical cancer
DAB: 3, 3’-diaminobenzidine
IHC: immunohistochemistry
NIH: National Institutes of Health
SUV: standardized uptake value
