# Correction: Screening of cancer tissue arrays identifies CXCR4 on adrenocortical carcinoma: correlates with expression and quantification on metastases using ^64^Cu-plerixafor PET

**DOI:** 10.18632/oncotarget.26214

**Published:** 2018-10-02

**Authors:** Ido D. Weiss, Lyn M. Huff, Moses O. Evbuomwan, Xin Xu, Hong Duc Dang, Daniel S. Velez, Satya P. Singh, Hongwei H. Zhang, Paul J. Gardina, Jae-Ho Lee, Liza Lindenberg, Timothy G. Myers, Chang H. Paik, David S. Schrump, Stefania Pittaluga, Peter L. Choyke, Tito Fojo, Joshua M. Farber

**Affiliations:** ^1^ Laboratory of Molecular Immunology, National Institute of Allergy and Infectious Diseases, National Institutes of Health, Bethesda, MD, USA; ^2^ Medical Oncology Branch, Center for Cancer Research, National Cancer Institute, National Institutes of Health, Bethesda, MD, USA; ^3^ Laboratory of Pathology, Center for Cancer Research, National Cancer Institute, National Institutes of Health, Bethesda, MD, USA; ^4^ Genomic Technologies Section, Research Technologies Branch, National Institute of Allergy and Infectious Diseases, National Institutes of Health, Bethesda, MD, USA; ^5^ Radiopharmaceutical Laboratory, Nuclear Medicine Division, Radiology and Imaging Sciences, Clinical Center, National Institutes of Health, Bethesda, MD, USA; ^6^ Molecular Imaging Program, Center for Cancer Research, National Cancer Institute, National Institutes of Health, Bethesda, MD, USA; ^7^ Thoracic Epigenetics Section, Thoracic and GI Oncology Branch, Center for Cancer Research, National Cancer Institute, National Institutes of Health, Bethesda, MD, USA

**This article has been corrected:** On page 73395, the following sentences have been updated to read, “Dosimetry for ^64^Cu-plerixafor calculated from this single patient gave an Effective Dose of 0.283 rem/mCi, and a total of 2.43 rem from the dose of 8.6 mCi. The organs that contributed the most to the Effective Dose were the liver and bone marrow (0.0606 and 0.0760 rem/mCi, respectively)”.

Similar to results in mice, the liver had the highest uptake of the tracer, with unbound tracer excreted through the kidneys [31, 32]. Significant uptake was also seen in organs of the immune system, including spleen, vertebral bodies (bone marrow), and lymph nodes (Figure 4 and Supplementary Figure 6). Of additional interest, uptake of ^64^Cu-plerixafor was absent from a number of vertebral bodies in the thoracolumbar spine that were within the region of prior radiation therapy (Figure 4 and Supplementary Figure 6). Dosimetry for ^64^Cu-plerixafor calculated from this single patient gave an Effective Dose of 0.283 rem/mCi, and a total of 2.43 rem from the dose of 8.6 mCi. The organs that contributed the most to the Effective Dose were the liver and bone marrow (0.0606 and 0.0760 rem/mCi, respectively). PET/CT sections (Figure 4B) showed variable uptake in the multiple pulmonary nodules.

Original article: Oncotarget. 2017; 8:73387-73406. https://doi.org/10.18632/oncotarget.19945

