# Supplementary material for: Screening of cancer tissue arrays identifies CXCR4 on adrenocortical carcinoma: correlates with expression and quantification on metastases using 64Cu-plerixafor PET
Source: Oncotarget. 2017 Aug 4;8(43):73387–406. doi: 10.18632/oncotarget.19945 (PMC5650270; doi:10.18632/oncotarget.19945)
Supplement: Supplementary file 1 [file oncotarget-08-73387-s001.pdf]

Screening of cancer tissue arrays identifies CXCR4 on adrenocortical carcinoma: correlates with expression and quantification on metastases using ^{64}Cu-plerixafor PET

SUPPLEMENTARY MATERIALS

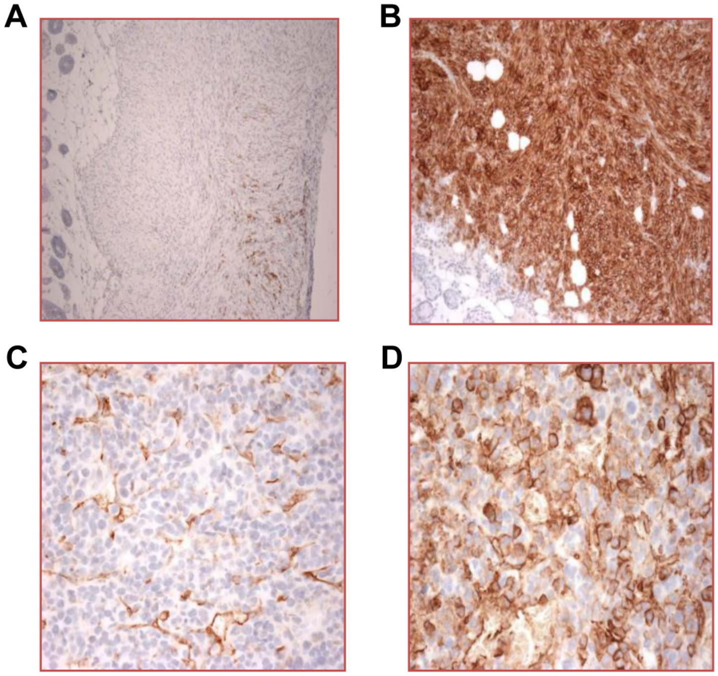

Supplementary Figure 1: Validation of CXCR4 detection in paraffin-embedded tissue by IHC of experimental tumors excised from mice. (**A**) CHO cell tumor (non-CXCR4-expressing tissue). Magnification is X 20. (**B**) CHO-XR4 tumor (high CXCR4-expressing tissue). Magnification is X 200. (**C**) 3LL Lewis lung carcinoma tumor (non-CXCR4-expressing cancer tissue, blood vessels staining positive). Magnification is X 400. (**D**) 3LL-XR4 tumor (low CXCR4-expressing tissue). Magnification is X 400. See Materials and Methods for details.

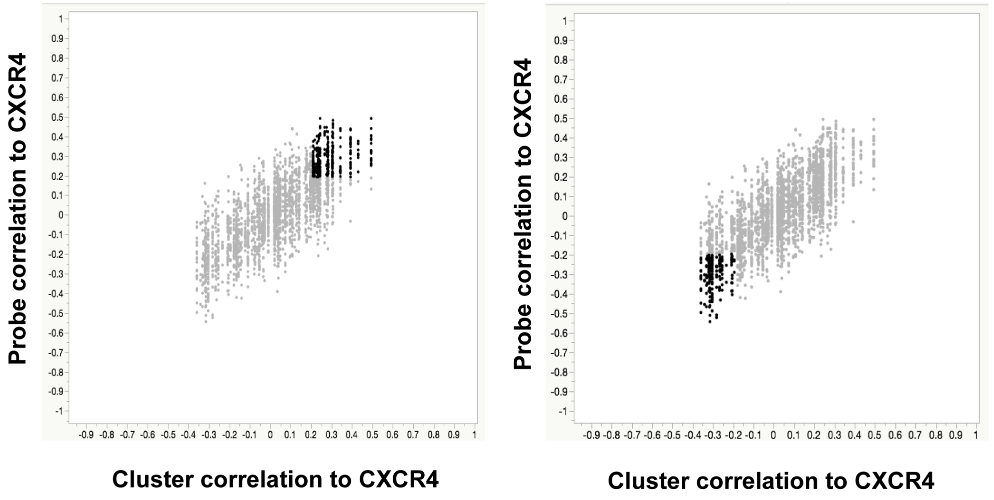

Supplementary Figure 2: Correlations of genes and gene clusters with expression of *CXCR4* in metastatic ACC. Genes from microarray analysis of 63 samples with normalized values for expression having standard deviations > 1.0 are plotted with respect to their correlation with *CXCR4* expression (Y-axis) versus the correlation with *CXCR4* expression for the centroid of the cluster to which they belong (X-axis). Positive- (left panel) and negative- (right panel) correlating probes that were analyzed further for Gene Ontology term enrichment are highlighted in black (see Supplementary Figures 3 and 4).

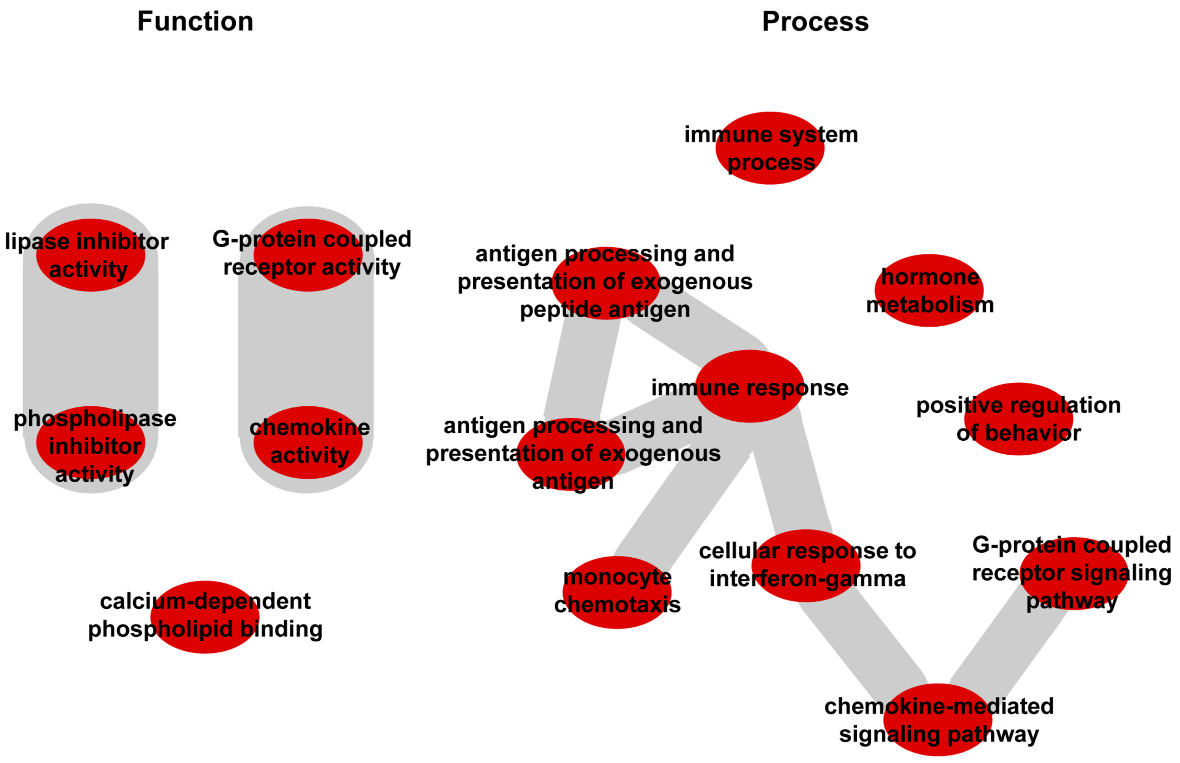

Supplementary Figure 3: Gene Ontology term enrichment analysis (see Materials and Methods) for gene probes having correlations with *CXCR4* expression of > 0.2 for both the probe itself and the probe's cluster (see Supplementary Figure 2).

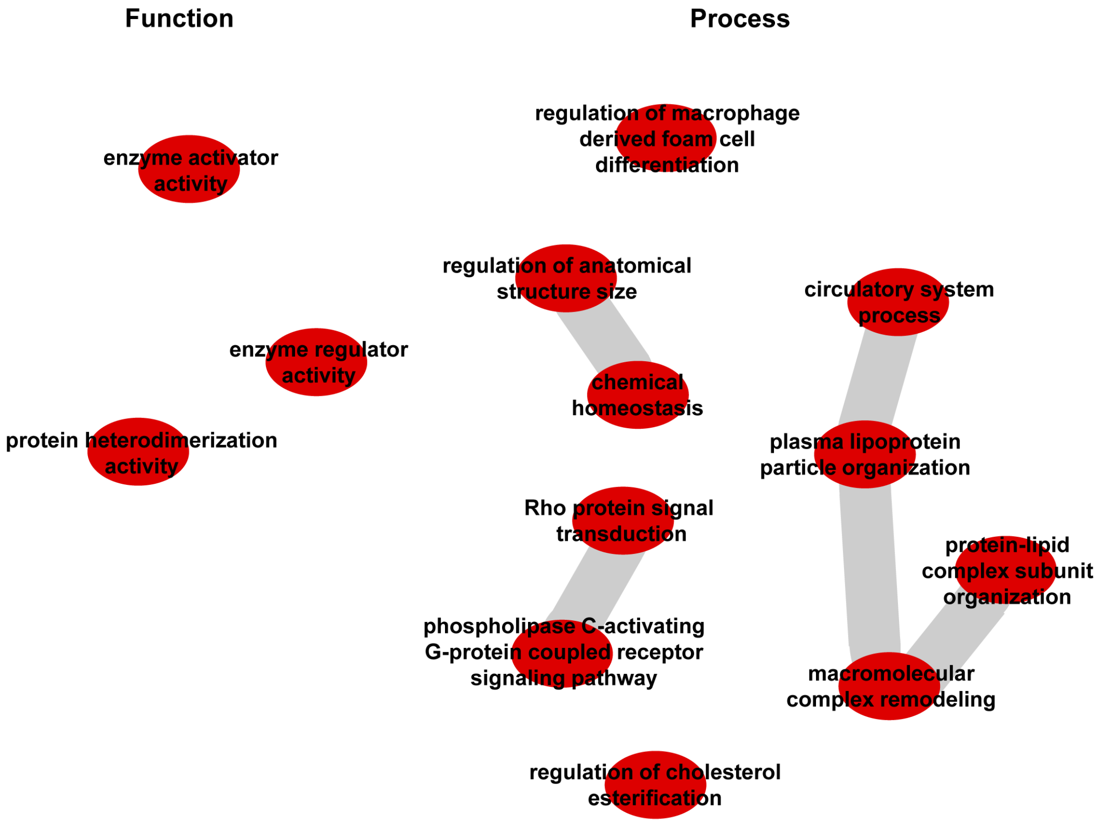

Supplementary Figure 4: Gene Ontology term enrichment analysis (see Materials and Methods) for gene probes having correlations with CXCR4 expression of < - 0.2 for both the probe itself and the probe's cluster (see Supplementary Figure 2).

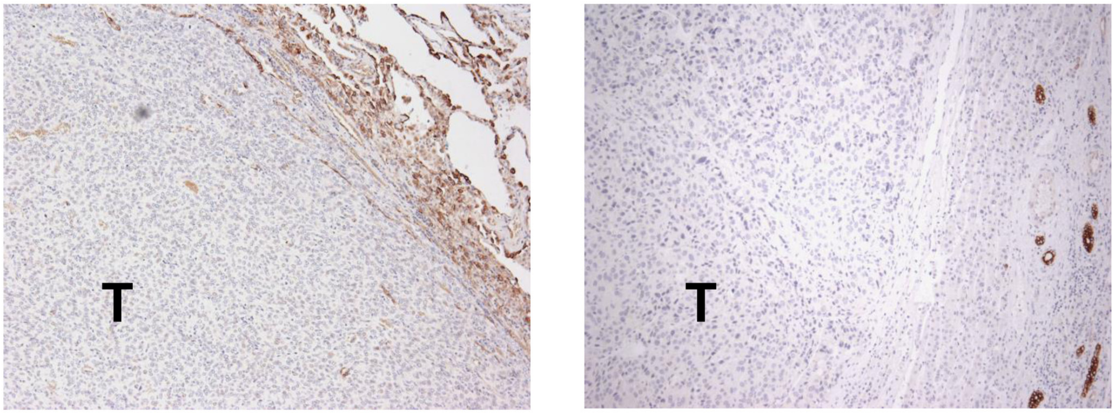

Supplementary Figure 5: Staining of ACC metastases for CXCL12. ACC metastases (T) in the lung (left) and liver (right), stained for CXCL12 using IHC with visualization using DAB (3, 3'-diaminobenzidine). Lung metastasis shows staining in tumor capillaries and adjacent lung parenchyma, and liver metastasis shows staining in adjacent bile ducts. Magnification is X 10. These same metastases were CXCR4$^+$ by IHC (data not shown). A total of 15 ACC metastases were stained similarly for CXCL12 and none showed CXCL12$^+$ cancer cells.

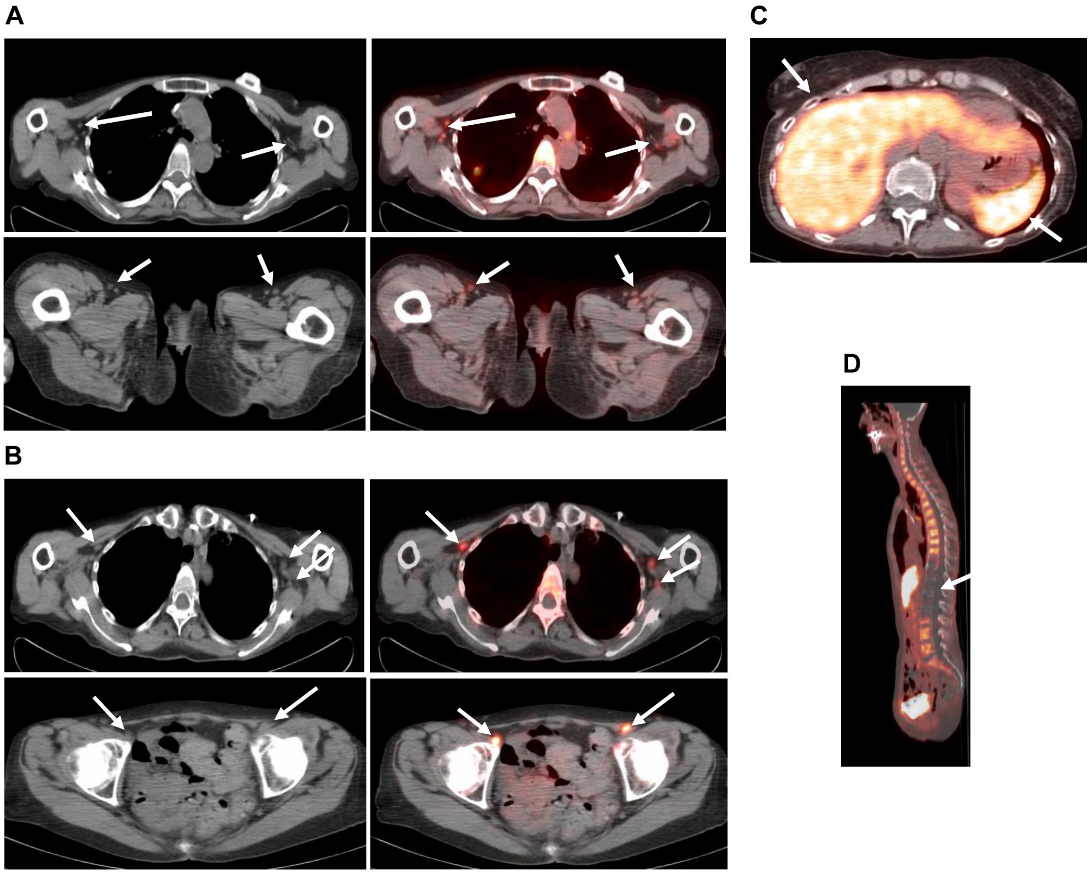

Supplementary Figure 6: ^{64}Cu-plerixafor uptake in normal tissues. (**A**) Transaxial plane CT (left) and fused CT and PET (right) images at the axillary (top) and femoral (bottom) levels 40 minutes following injection of ^{64}Cu-plerixafor showing uptake of ^{64}Cu-plerixafor by normal lymph nodes as indicated by the arrows. (**B**) Transaxial plane CT (left) and fused CT and PET (right) images at the axillary (top) and inguinal (bottom) levels 23 hours following injection of ^{64}Cu-plerixafor showing uptake of ^{64}Cu-plerixafor by normal lymph nodes as indicated by the arrows. (**C**) Transaxial plane fused CT and PET images 40 minutes following injection of ^{64}Cu-plerixafor showing uptake of ^{64}Cu-plerixafor by the liver (upper left) and spleen (lower right) as indicated by the arrows. (**D**) Sagittal plane fused CT and PET images 40 minutes following injection of ^{64}Cu-plerixafor showing uptake of ^{64}Cu-plerixafor by vertebral bodies above and below an area of prior radiation therapy indicated by the arrow.

Supplementary Table 1: Chemokine and chemokine receptor genes correlating with *CXCR4* expression in metastatic ACC

Gene	Protein
CXCL2	chemokine (c-x-c motif) ligand 2
CXCL3	chemokine (c-x-c motif) ligand 3
CXCL8	chemokine (c-x-c motif) ligand 8 (IL-8)
CXCL13	chemokine (c-x-c motif) ligand 13
CCL2	chemokine (c-c motif) ligand 2
CCL4	chemokine (c-c motif) ligand 4
CCL5	chemokine (c-c motif) ligand 5
CCL8	chemokine (c-c motif) ligand 8
CCL18	chemokine (c-c motif) ligand 18
CCL19	chemokine (c-c motif) ligand 19
CCL20	chemokine (c-c motif) ligand 20
CCL21	chemokine (c-c motif) ligand 21
ACKR3	atypical chemokine receptor 3 (CXCR7)
CX3CR1	chemokine (c-x3-c motif) receptor 1
